# Intra-hospital MRI transport in neurocritical patients with aneurysmal subarachnoid hemorrhage: complications and clinical impact with predominant continuation of care

**DOI:** 10.1007/s10143-025-03824-3

**Published:** 2025-09-26

**Authors:** Alexandra Grob, Jonas Georg Buff, Lilian Kriemler, Federica Stretti, Giovanna Brandi

**Affiliations:** 1 Institute for Intensive Care Medicine, University Hospital Zurich, University of Zurich, Zurich, Switzerland; 2 Department of Neurosurgery, University Hospital Zurich Clinical Neuroscience Center, University of Zurich, Rämistrasse 100, Zurich, 8091 Switzerland

**Keywords:** Intracranial pressure, MRI, Intrahospital transport, Cerebral perfusion pressure, Neuromonitoring, Neurocritical patients, Subarachnoid hemorrhage

## Abstract

Introduction Magnet Resonance Imaging (MRI) is essential for neurocritical care but requires intrahospital transport (IHT) in patients treated in an intensive care unit, which carries significant risks. This study assesses the risk profile and whether the benefits of MRI in aneurysmal subarachnoid hemorrhage (aSAH) outweigh the associated complications of IHT. Method In this retrospective study, all aSAH patients treated in our neurocritical care unit (NCCU) between 2016 and 2023 were screened. Data collection included baseline demographics, hemorrhage severity scores, aneurysm treatment strategies, additional neurosurgical procedures, and need for spasmolysis. IHT- and MRI-related variables were recorded, including timing, indication, duration, and associated physiological parameters such as blood gas values, vital signs, intracranial pressure (ICP), and cerebral perfusion pressure (CPP). The incidence of complications and the clinical impact of MRI findings were evaluated. Statistical comparisons were conducted using the Wilcoxon signed-rank test. Results Of 337 screened patients, 115 (34.1%) patients with aSAH underwent a MRI during the NCCU stay and were included in the analysis, with a total of 156 MRI scans performed. The most common reason for a MRI was ischemia exclusion (61%). Complications occurred in 16% of patients (*n* = 25), classified as systemic (56%), cerebral (37%), and technical (7%). Blood gas analysis showed no significant changes before and after IHT. In the subgroup of patients with an external ventricular drain (EVD) in situ (*n* = 93), ICP remained stable, while CPP increased from 85.4 (± 20.5) to 92.1 (± 19.9) mmHg (*p* = 0.009). Accordingly, systolic BP and MAP increased (*p* = 0.019 and < 0.001, respectively) and HR decreased by 4/min (*p* < 0.001). Conclusion We found lower frequency of IHT-related complication in brain MRI after aSAH compared to existing literature reflecting a well-established and safe clinical process. Moreover, MRI findings had a notable impact on key therapeutic decisions, supporting the use of MRI-based IHT in selected cases, provided that a thorough risk-benefit assessment is conducted.

## Introduction

Neurocritical care monitoring, including intracranial pressure (ICP) measurement, cerebral perfusion pressure (CPP) assessment, and advanced multimodal monitoring, has significantly improved; nevertheless, neuroimaging remains essential for the diagnosis, monitoring, and management of neurocritically ill patients. The role of cerebral computed tomography (CT) and magnetic resonance imaging (MRI) is well-established and crucial for diagnosing hemorrhage, ischemia, hydrocephalus, or secondary ischemic events [1] in patients with acute brain injury, as well as aiding tool in prognostic assessment [2]. However, MRI presents challenges due to its higher cost, limited availability, prolonged scanning duration and incompatibility with ferromagnetic materials.

Both CT and MRI require intrahospital transports (IHT), which can lead to a high frequency of complication [1], particularly in critically ill patients treated at the neurocritical care unit (NCCU) requiring several medical devices, such as mechanical ventilation. Transport-related complications can affect systemic and cerebral parameters and lead to technical device dysfunctions, potentially necessitating urgent intervention. For instance, an acute increase in ICP can reduce CPP, and hence increase the risk of secondary brain injury [3, 4]. Moreover, the ability to perform immediate interventions is limited both during IHT and within the MRI suite. Beyond medical risks, IHT for MRI demands extensive logistical coordination, including specialized personnel, pre-transport checklists, and adherence to strict safety protocols to ensure patient stability before, during, and after imaging [5, 6].

MRI is an essential diagnostic tool for aneurysmal subarachnoid hemorrhage (aSAH) patients in the NCCU, as it provides more sensitive detection of secondary ischemic injuries compared to CT, which often fails to identify early or small infarcts [7]. Diffusion-weighted imaging (DWI) allows for the early identification of ischemic lesions, which carry significant prognostic value and can influence therapeutic strategies [8]. Additionally, MRI can detect microvascular changes and inflammatory processes in brain tissue that are not visible on CT [9]. Particularly in cases of unexplained neurological deterioration despite unremarkable CT findings, MRI is necessary to assess for delayed cerebral ischemia (DCI) or hydrocephalus with greater detail, thereby supporting more targeted clinical decision-making [10].

Due to the high proportion of poor-grade aSAH patients, many require deep sedation during the early phase of treatment [11]. Consequently, routine CT imaging is often performed to detect vasospasm or DCI, with IHT being an essential part of patient management [12]. Despite this, the actual therapeutic impact of neuroimaging remains uncertain. Previous studies analyzing diverse neurosurgical populations have reported that the rate of imaging findings leading to therapeutic consequences ranges widely from 3 to 66% [13–16]. This considerable variation raises the question of whether alternative bedside monitoring techniques could provide similar diagnostic insights without the risks associated with IHT.

This retrospective study investigates the frequency and type of complications that arose during IHTs of patients with aSAH. It also evaluates whether the additional information provided by MRI has led to therapeutic changes in patient management, to assess whether the possible risks associated with imaging justify the additional diagnostic and prognostic information gained by MRI.

## Method

### Study design and population

We conducted a retrospective observational cohort study. All patients aged ≥ 18 years who were admitted to the NCCU at University Hospital Zurich, Switzerland, with aSAH between January 2016 and December 2023 were screened for eligibility. Inclusion criteria were: (1) Adult patients with aSAH; (2) who underwent at least one MRI during their NCCU-stay. Patients were considered, regardless of the respiratory support (spontaneously breathing, intubated, or tracheotomized). The diagnosis of aSAH was confirmed by presence of a ruptured aneurysm on CT angiography (CTA) or subsequent digital subtraction angiography (DSA). Exclusion criterium was patient’s written or documented oral refusal to have their data analyzed for research projects.

The study was conducted in accordance with the ethical guidelines of the Canton of Zurich and approved by the local ethics committee of Zurich (KEK: 2023 − 01046).

### Image acquisition

All MRI examinations were performed using a standardized protocol on a 3 T scanner. The routine imaging protocol included the following sequences: DWI, axial T2-weighted imaging, susceptibility-weighted imaging (SWI), 3D FLAIR, time-of-flight (TOF) MR angiography of the intracranial arteries, and pre- and post-contrast T1-weighted 3D MPRAGE sequences. In addition, a 3D phase-contrast angiography sequence was acquired to assess venous sinuses and cerebral veins. Gadolinium-based contrast agent (Dotarem^®^) was administered intravenously when indicated. Scan time was less than 60 min.

### Patient’s population and management

Patients were treated based on the last available guideline for management of patients with aSAH. In case of hydrocephalus/ventriculomegaly, an external ventricular drain (EVD) was inserted [17]. Over a period of at least 14 days, patients were treated at the NCCU or at the intermediate care unit due to the risk of vasospasm/DCI. In case of a prolonged unconscious state, either due to the severity of the disease itself or due to the necessity for deep sedation as part of the diagnostic and therapeutic management, an invasive multimodal neuromonitoring (MMN) including cerebral microdialysis (CMA 70, CMA Mikrodialysis, Solna, Sweden), brain tissue oxygenation monitoring (LiCox system, Integra Neurosciences, Plainsboro, NJ), and continuous electroencephalography was inserted. In all patients with MMN, the monitoring devices were removed prior to MRI due to technical limitations. Daily bedside transcranial doppler (TCD) examinations were systematically performed and served as a key component in the interdisciplinary decision-making process regarding the indication for MRI and removal of the MMN.

At our institution, once a patient is deemed transportable—regardless of intubation status or vasopressor requirement—the decision between CT and MRI is made on diagnostic grounds. In case of MRI, patients were transported in their own beds to the neuroradiology department. The neurology department is located on the same floor as the NCCU. An experienced NCCU-resident, accompanied by an NCCU-nurse and a nursing assistant, escorts the patient throughout the entire IHT. The physician remains present with the patient in the MRI suite for the duration of the scan.

Transport preparation was conducted bedside, including review of a standardized checklist that includes clearance for e.g. cardiac pacemaker or implants. Continuous monitoring of heart rate (HR), blood pressure, respiratory rate, peripheral arterial oxygen saturation (sO2) and, if available, ICP and CPP was maintained throughout the transport. An emergency kit containing essential medications was always carried during IHT from the NCCU to the MRI suite.

### Outcome variables and measurements

Patient data were extracted from electronic medical records, including KISIM™ (*Cistec AG*,* Zurich*,* Switzerland*) and MetaVision (*iMDsoft*,* Tel Aviv*,* Israel*). The following parameters were collected: (1) Baseline patient characteristics, including age, sex, and clinical presentation; (2) aSAH severity grading scores, including initial Glasgow Coma Scale (GCS), Hunt & Hess scale [18], Fisher grading scale [19], and World Federation of Neurosurgical Societies (WFNS) scale, being dichotomized into “good” (grades 1–3) and “poor” (grades 4–5) [20]; (3) Aneurysm treatment modalities (neurosurgical or neurovascular treatment); (4) need and type of neurosurgery, including hematoma evacuation, decompressive hemicraniectomy, EVD placement, MMN placement; (5) occurrence of vasospasm and the need for intraarterial spasmolysis. Furthermore, we analyzed IHT-related variables, including: (1) Timing of MRI (expressed as number of days after aSAH); (2) reason for MRI (exclusion of vascular dissection, ischemia, above all brain stem ischemia, search for or reperfusion of a treated aneurysm or postoperative infection/abscess); (3) need of airway and respiratory devices (endotracheal tube, tracheostomy; spontaneous breathing or mechanical ventilation) and ventilation mode; (4) medications administered during IHT; and (5) vital parameters, including:

- Systemic parameters: Mean arterial pressure (MAP), systolic blood pressure (SBP), heart rate (HR), and respiratory rate (RR).
- Intracranial parameters: ICP and CPP.
- Blood gas analysis (BGA): pO₂, pCO_2_, pH, lactate (Lac).

We compared BGAs performed before (until 1 h) and after (within 3 h) IHT. Vital parameters were recorded 1 h before, as well as 1 and 2 h after IHT. If an EVD was in place, intracranial parameters were considered 1 h before, during, and 1 h after IHT. Average value was determined based on visual average of the time course data with exclusion of outliers. As an outcome measure, the Glasgow Outcome Scale-Extended (GOS-E) was assessed at 3 and 12 months after hemorrhage; for subgroup analyses, GOS-E scores were dichotomized into favorable and unfavorable outcomes, with an unfavorable outcome defined as a score of ≤ 3 [21–25].

### Complications and consequences

Complications were classified into three categories: systemic, cerebral, and technical. The clinical consequences of each complication were analyzed. Systemic complications included sO2 desaturation below 90%, changes in blood pressure requiring drugs administration (e.g. catecholamines or antihypertensive drugs), bleeding or leakage at the site of any medical device/catheter with need of intervention/replacement, and allergic reactions to gadolinium. Cerebral complications were defined as an ICP increase > 20 mmHg, or a CPP < 60mmHg needing an intervention (e.g. CSF drainage), bleeding/leak at the EVD skin insertion site or disconnection of the EVD during or after IHT. Technical complications encompassed e.g. malfunctions of medical devices, such as infusion pumps or the depletion of oxygen cylinders during IHT; dislocation of vascular lines, as central venous catheter or arterial catheter.

For each MRI, the clinical consequences were further evaluated, including indications for additional diagnostics such as DSA, need of surgical interventions, changes in CSF management (EVD drainage volume, removal of the EVD, change of shunt settings), adaption in medical treatment or change in treatment goal, e.g. redirection of care to palliation.

### Statistical analysis

Systemic and intracranial parameters were compared before and after IHT. Continuous variables are presented as mean ± standard deviation (SD). Categorical variables are expressed as median and interquartile range (IQR). Differences in variables before and after IHT for MRI were analyzed using the Wilcoxon matched-pairs signed-rank test, since our data is not normally distributed. Subanalysis for complication rate and functional outcome at 12 months was performed with one-sided Fisher’s exact test. A p-value < 0.05 was considered statistically significant. Statistical analyses were conducted using Python (Python Software Foundation, *Python Language Reference*, version 3.11.9, Wilmington, Delaware, USA).

## Results

### Patient data and clinical characteristics

Overall, 337 patients were admitted to the NCCU with an aSAH during the study period, of whom 115 (34.1%) underwent MRI during the NCCU stay. In total, 156 MRI scans were performed (36% of the patients had at least 2 scans). Examples for repeated MRI included: assessment of posterior fossa ischemia or evolving pseudoaneurysms (e.g., PICA), follow-up after incomplete DSA due to suspected vertebral artery dissection or unclear aneurysm source, exclusion of thromboembolic complications post-DSA, evaluation of ventriculitis or postoperative infection or intracranial/intraventricular abscess, early MRI for treatment planning in multiple aneurysms and follow-up for ischemic or hypoxic brain damage, dedicated vessel wall imaging for source identification or ischemia after spasmolysis, planned monitoring of partially thrombosed aneurysms (e.g., basilar tip), and artifact-reducing follow-ups in cases with extensive endovascular coiling material.

Baseline characteristics, location of ruptured aneurysms, severity scores, and radiological findings on first head-CT are presented in Table 1. Most patients were female (*n* = 103, 66%). The mean age was 57.3 (± 12.8) years. A total of 93 (60%) patients received an EVD. IHT-related data, including missing values, are presented in Table 2. The cn MRI was exclusion of ischemia (*n* = 110, 61%).

**Table 1:** Baseline characteristics

Parameter	*N* = 156
Age [years]	57.3 (± 12.8)
Sex Female	103 (66.0%)
NCCU-LOS [days]	22 (± 14.5)
Hospital LOS, [days]	35.6 (± 52.0)
Patients died in NCCU (due to redirection of care to palliation)	21 (13.5%)
Aneurysm location ICA MCA ACA VA BasilarA SCA PcomA AcomA PericallosalA PICA ChoroidAntA	15 (9.6%) 29 (18.6%) 2 (1.3%) 12 (7.7%) 17 (10.9%) 1(0.6%) 31 (19.8%) 25 (16.0%) 2 (1.3%) 18 (11.5%) 3 (2%)
Multiple aneurysms Yes	58 (37.2%)
WFNS Grading Grade 1 Grade 2 Grade 3 Grade 4 Grade 5	53 (33.9%) 28 (17.9%) 8 (5.1%) 29 (18.6%) 38 (24.4%)
Hunt & Hess Grading Grade 1 Grade 2 Grade 3 Grade 4 Grade 5	31 (19.8%) 37 (23.7%) 33 (21.2%) 25 16.0%) 30 (19.2%)
Fisher Grading Grade I Grade II Grade III Grade IV	10 (6.4%) 11 (7.1%) 61 (39.1%) 74 (47.4%)
Intracerebral Bleeding Yes	39 (25.0%)
Subdural Hematoma Yes	20 (12.8%)
Hydrocephalus Yes	89 (57.1%)
Blood in basal cisterns Yes	107 (69.2%)
Intraventricular hemorrhage Yes	112 (71.8%)
Treatment modality Endovascular coiling or stenting Surgical therapy (e.g. clipping, trapping) Conservative	86 (55.1%) 68 (43.6%) 2 (1.3%)
Cerebrospinal fluid drainage None External ventricular drainage	63 (40.4%) 93 (59.6%)
Multimodal neuromonitoring Yes	32 (21.5%)
Intraarterial spasmolysis Yes	47 (30.1%)

Continuous variables are presented as mean ± standard deviation (SD), while categorical variables are expressed as median ± interquartile range (IQR). Abbreviations: *LOS* length of stay, *MRI* magnet resonance imaging, *NCCU* neurointensive care unit, *ICA* Internal Carotid Artery, *MCA* Middle Cerebral Artery, *AcomA *Anterior communicating Artery, *PericallosalA* Pericallosal Artery, *ACA *Anterior Cerebral Artery, *VA *Vertebral Artery, *PCA *Posterior Cerebral Artery, *PcomA* Posterior communicating Artery, *BA* Basilar Artery, *PICA* Posterior Inferior Cerebellar Artery, *WFNS *World Federation of Neurosurgical Societies Score

**Table 2:** IHT-related parameters and complications

Parameter	
Transports performed	156 (100%)
Timepoint of MRI after admission [days]	8.9 (± 9.5)
Reason for MRI* Exclusion of vascular dissection Ischemia Reperfusion of aneurysm Search for aneurysm Abscess Other	6 (3.3%) 110 (61.1%) 26 (14.4%) 19 (10.6%) 4 (2.2%) 15 (8.3%)
Airway and respiratory support None Endotracheal tube Tracheostomy Support with high-flow oxygen	54 (34.6%) 83 (53.2%) 18 (11.6%) 1 (0.6%)
Transportation Time [min]	109 (± 30.7)
Transport medication including baseline sedation None Propofol Muscle relaxant, e.g. Rocuronium Catecholamins, e.g. Noradrenaline Antihypertensive agents, e.g. Clevidipine Opioids, e.g. Fentanyl Electrolyte solution Anesthetics, e.g. Ketamine Benzodiazepine, e.g. Midazolam alpha-2 receptor agonist, e.g. Clonidin	15 (6.0%) 69 (27.6%) 8 (3.2%) 61 (24.4%) 16 (6.4%) 58 (23.2%*)* 1 (0.4%) 4 (1.6%) 13 (5.2%) 5 (2.0%)
Overall complication frequency Yes	27 (17.0%)
Systemic Complications* SpO2 < 90% BP change requiring medical intervention Arrhythmia Allergic reaction to Gadolinium	15 (55.6%) 3 (11.1%) 8 (29.6%) 1 (3.7%) 3 (11.1%)
Cerebral Complications* ICP > 20mmHg Seizures Wet wound at surgical site/Bleeding	10 (37.0%) 8 (29.6%) 0 (0.0%) 2 (7.4%)
Technical Complications* Catheter replacement needed	2 (7.4%) 2 (7.4%)
Consequence of diagnostic MRI “None” Surgery Dual subtraction angiography (diagnostic or intervention) Additional medical established treatment Change of therapy goal Change of CSF drainage amount Removal of CSF drainage device Change of VP shunt settings	104 (66.7%) 13 (8.3%) 11 (7.1%) 7 (4.5%) 8 (5.1%) 8 (5.1%) 3 (1.9%) 2 (1.3%)

Continuous variables are presented as mean ± standard deviation (SD). * Multiple complications for the same patient are possible. Abbreviations: *IHT *intrahospital transport, *MRI* magnetic resonance imaging, *BP *blood pressure, *ICP* intracranial pressure, *CSF* cerebrospinal fluid, *VP* ventriculoperitoneal

Twenty-five patients (16%) had at least one complication. The total number of complications was 27, with 56% (*n* = 15) classified as systemic, 37% (*n* = 10) as cerebral, and 7% (*n* = 2) as technical complications. Table 2 shows detailed.

Overall MRI findings (Table 2) led to no change in clinical management in most cases (67%), with 63% of them being classified with a “good” WFNS, indicating continuation of the existing treatment strategy. In 8% of patients, the imaging results prompted surgical intervention, while 7% underwent DSA for further diagnostic or therapeutic purposes. A change in therapy goals was documented in 5% of cases, whereas 75% of them had a “poor” WFNS.

The subanalysis for the complication rate according to “good” and “poor” WFNS grading or intubation status does not show any difference. While worse clinical outcome with GOS-E (grading > 5) after 12 months correlates with the severity of bleeding (WFNS grade 4–5) and ventilation mode (intubated).

**Fig. 1:**
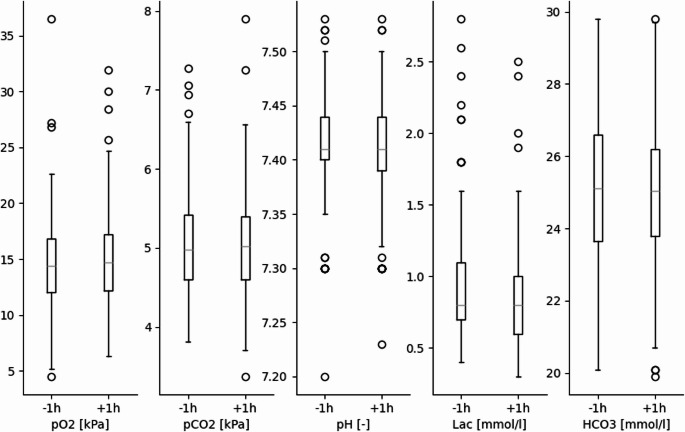
Boxplot development blood gas analysis before and after transport without significant differences. Units presented on y axis, stated in square brackets

**Fig. 2:**
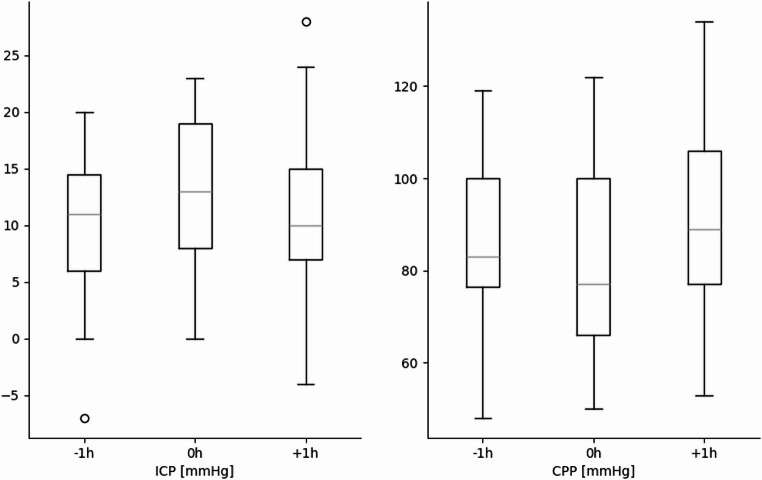
Boxplot development ICP and CPP before and after transport. Significant increase of CPP after IHT (*p* = 0.009). Units presented on y axis, stated in square brackets

**Fig. 3:**
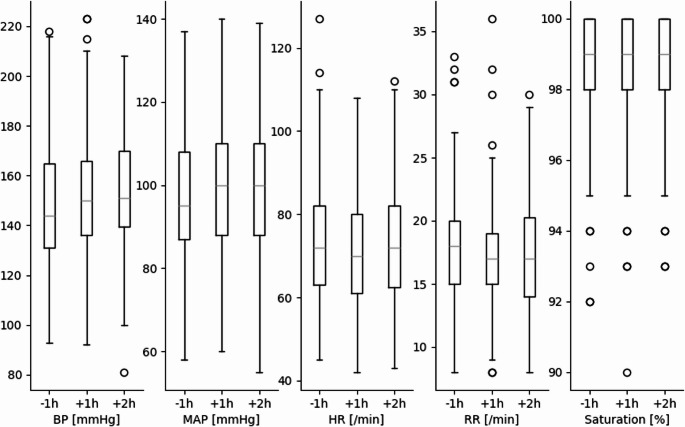
Boxplot development vital signs before and after transport. Significant increase of BP one and two hours after transportation (*p* = 0.019 and 0.204) and significant increase of MAP and HR one hour before and after (both *p* < 0.001). Units presented on y axis, stated in square brackets

BGA parameters are illustrated in Fig. 1; Table 3 showing no significant changes before and after IHT. In patients with an EVD (*n* = 93; 60%), there was no difference in ICP values measured before, during, or after IHT. However, CPP was higher 1 h after compared to 1 h before transportation from 85.4 (± 20.5) to 92.1 (± 19.9) mmHg (*p* = 0.009) (Fig. 2; Table 3). Vital parameters before and after IHT showed a significant increase in systolic BP and MAP from 149 (± 24.8) to 152 (± 25.9) mmHg and from 97 (± 15.3) to 100 (± 17.0) mmHg (*p* = 0.019 and < 0.001, respectively; Fig. 3). HR was significantly lower one hour after transportation by 4/Min (*p* < 0.001).

**Table 3:** Outcome variables before, during and after IHT in aSAH patients with MRI while being treated in the NCCU

Parameter	1 h before	during	1 h after	2 h after	*p*-value
pO2 [kPa]	14.6 (± 4.0)		15.3 (± 4.6)		0.587 (*n* = 146)
pCO2 [kPa]	5.05 (± 0.6)		5.04 (± 0.7)		0.982 (*n* = 147)
pH	7.42 (± 0.047)		7.41 (± 0.048)		0.824 (*n* = 147)
Lactate [mmol/L]	0.951 (± 0.417)		0.852 (± 0.357)		n.a. (*n* = 147)
HCO3 [mmol/L]	25 (± 2.5)		25 (± 2.0)		0.773 (*n* = 144)
ICP [mmHg]	10.2 (± 5.8)	12.6 (± 7.5)	10.5 (± 6.7)		0.106 (*n* = 21) 0.605 (*n* = 70)
CPP [mmHg]	85.4 (± 20.5)	82.7 (± 22.8)	92.1 (± 19.9)		0.778 (*n* = 19) 0.009 (*n* = 66)
Systolic BP [mmHg]	149 (± 24.8)		152 (± 25.9)	154 (± 23.4)	0.019 (*n* = 150) 0.0204 (*n* = 147)
MAP [mmHg]	97 (± 15.3)		100 (± 17.0)	98.4 (± 16.3)	< 0.001 (*n* = 145) 0.157 (*n* = 141)
Heart rate [/Min]	74 (± 14.5)		70.7 (± 12.8)	73.7 (± 14.4)	< 0.001 (*n* = 150) 0.882 (*n* = 147)
Respiratory rate [/Min]	18 (± 4.3)		17.2 (± 4.4)	17.3 (± 4.7)	0.133 (*n* = 129) 0.299 (*n* = 127)
Saturation [%]	98.5 (± 1.7)		98.7 (± 1.8)	98.6 (± 1.7)	0.423 (*n* = 148) 0.795 (*n* = 143)

Continuous variables are presented as mean ± standard deviation (SD). A p-value < 0.05 was considered statistically significant. First p-value are parameters compared 1 h before and 1 h after, second p-value is 1 h before and during or 2 h after, respectively. Abbreviations: *aSAH* acute subarachnoid hemorrhage, *IHT* intrahospital transport, *MRI* magnet resonance imaging, *NCCU* neurointensive care unit, *ICP* intracranial pressure, *CPP* cerebral perfusion pressure, *BP* blood pressure, *MAP *mean arterial pressure

## Discussion

In this paper, we analyzed the timing, frequency and type of relevant IHT related complications in patients with aSAH who underwent an MRI during the NCCU-stay. Additionally, we evaluated the changes in medical decisions/management and the need of further interventions based on the MRI-findings. As a key finding, the rate of IHT complications in our population is relatively low. Moreover, the new information obtained from MRI induced changes in treatment management in one third of the patients, a proportion that reflects a relevant diagnostic and therapeutic impact in this critically ill population.

The relatively low frequency of IHT-related complications suggests that well-structured workflows and highly trained personnel seem to be essential for safe transport. The first guidelines for IHT were published in 1993 [26] and were last updated in 2004 [27]. Given the increasing use of advanced diagnostic modalities in NCCUs [28], the current guidelines may no longer adequately reflect present-day clinical demands. In response, checklists have been introduced to enhance patient safety during IHT [27, 29–31]. Many of the reported complications of the study population (56%) were systemic, primarily involving an increase in blood pressure, which required administration of sedatives and/or antihypertensive therapy and could therefore be treated promptly.

Ensuring patient safety during IHT for MRI is critical, with several studies identifying key risk factors and monitoring parameters to maintain physiological stability. Schmidbauer et al. were the first to propose specific thresholds based on expert opinion and retrospective data, though these have not yet been prospectively validated [32]. When applying their criteria to our cohort, we observed only 6% of cases exceeding neurological thresholds (ICP > 22 mmHg), while we argue that systolic BP > 180 mmHg should not be classified as a complication in aSAH patients, given its role in preventing vasospasm and DCI [33]. In our study, vital signs, blood gas values, and ICP remained largely stable before and after transport, likely reflecting the high level of neurocritical care expertise and the focus on MRI-specific IHTs. While Schmidbauer’s safety limits remain unvalidated [32], elevated ICP is widely acknowledged as a risk factor for reduced CPP and DCI. Prior studies have demonstrated that IHT can provoke ICP elevations, particularly in patients with already elevated baseline levels [3, 4, 13, 34], and that use of elevators or ramps may further exacerbate this and alter cerebral metabolism [4]. Additionally, the recommended 30° head-up positioning for optimal venous drainage and ICP reduction cannot be maintained during MRI [35].

Pinggera et al. investigated IHT for early MRI (median day 6) in a cohort of ventilated patients with acute, severe traumatic brain injury [36]. Their monitoring relied solely on end-tidal CO₂—a known surrogate with limitations compared to direct PaCO₂ measurement [37]—as well as MAP and ICP. While they reported stable ICP and MAP before and after transport, a significant decrease in end-tidal CO₂ up to 45 min after transport was recorded. Although they argue that these findings lack clinical relevance, the true impact remains rather uncertain—particularly with regard to potential hypocapnia-induced cerebral vasoconstriction and its effect on cerebral perfusion [38, 39], which was not directly measured. However, they highlight how important the coordination of involved teams (e.g. radiology and anesthesia) is along with the requirement for patients to be hemodynamically stable for four hours.

These observations further underscore the critical importance of evidence-based standard operating procedures (SOPs) in the management of high-risk patients. The study by Cuschieri et al. demonstrated that the implementation of SOPs in severely injured patients led to a significant reduction in mortality from 22 to 11% [40], highlighting that even partial adherence can improve outcomes—despite the inherent challenges in full protocol compliance. Although their cohort did not include patients with traumatic brain injury, the findings emphasize the broader value of structured protocols. An early example for this was proven Weg et al. in 1989, who concluded that manual ventilation during intrahospital transport was as safe when performed by trained personnel with defined protocols as mechanical ventilation [41]. However, this study also did not assess intracranial parameters, which are particularly sensitive in neurocritical care patients. Taken together, these findings [40, 41] highlight that clinical safety cannot rely solely on advanced technology; rather, it depends on well-trained staff capable of managing both equipment and unexpected complications in adherence to validated clinical standards.

Interestingly, nearly half of Pinggera et al.’s cohort underwent more than one MRI [36] whereas we count 36%. In our cohort, repeated MRIs were primarily used to improve diagnosis compared to CT (to investigate abscesses, partial thrombosis, residual perfusion of an aneurysm, or artifacts from endovascular coils). Their implications—such as suggested prolonged intubation for better functional outcome—were not evaluated [36].

Following MRI and interdisciplinary review, there was a change in our medical management in one third of the patients, comparable to the data reported by Schmidbauer et al., where the authors specifically analyzed IHT in SAH patients who underwent a head-CT scan and they reported that imaging findings directly influenced clinical decisions in 40% of cases. However, “no consequence” may still imply that ongoing therapy was continued and therefore had a clinical impact, which is significantly different from “no consequence at all” in clinical decision-making. In 5% of our cases the interdisciplinary decision agreed to change therapy goal based on the MRI results with most of them having a poor grade WFNS classification.

While CT remains the first-line modality in acute neurocritical care, MRI provides significantly greater sensitivity for detecting early ischemic changes, small infarcts, and posterior fossa lesions - areas where CT often underperforms due to beam-hardening artifacts and limited resolution due to anatomical complexity [42, 45]. In our cohort, MRI findings frequently led to specific and clinically relevant management changes that would not have been initiated based on CT alone. For example, early DCI-related infarcts detected on DWI [46] prompted the implantation of multimodal neuromonitoring; confirmation of a cerebral abscess following aneurysm clipping led to revision surgery [47]; and vessel wall imaging revealed residual perfusion or incomplete exclusion of aneurysms, prompting further surgical or endovascular treatment [48]. Particularly in posterior fossa strokes—where CT sensitivity is notably low—MRI proved essential for accurate diagnosis and therapeutic guidance, especially using DWI, which outperforms CT and CT perfusion in this region [43, 44]. Therefore, while MRI is not universally required, it provided critical diagnostic and therapeutic value in selected high-risk patients.

Martin et al. demonstrated a significant correlation between adverse events during IHT and prolonged NCCU stays in patients with traumatic brain injury undergoing IHT for CT imaging [13]. Technical complications during IHT have been reported at rates up to 46% [49, 50]. This frequency was lower in our population, but this is probably due to the lack of a standardized definition for technical failures and challenges in retrospective data collection, which makes direct comparisons difficult.

While literature reports 17–42% of IHTs as emergencies [4, 14, 32], in our study, MRI was mostly performed electively, usually on the same day of indication, after interdisciplinary discussion which allowed for safe preparation of the IHT. In emergency cases, CT was preferred for rapid decision-making. Given the diagnostic value of MRI, portable ultra-low-field MRI may offer a promising alternative, avoiding IHT-related risks [28, 51]. However, its lower image resolution, increased artifact susceptibility, and potential interference with life-sustaining NCCU equipment remain important limitations [52]. Due to the limitations of portable MRIs, we believe the focus should be on selecting patients for a safe transport and for whom the result of the imaging would impact the clinical management.

This study has several limitations. First, its retrospective and single-center design without a control group limits generalizability. Furthermore, all MRIs were performed outside the acute phase of aSAH, which may have influenced both complication rates and diagnostic yield. The most significant methodological limitation is that only single time-point values for intracranial and blood pressure parameters were analyzed, rather than averaged values over defined intervals (e.g., 5-minute means), which may have reduced the sensitivity for detecting transient but clinically relevant fluctuations. Another key limitation lies in the potential for selection bias. However, it is important to note that our cohort includes all consecutive patients who received MRI, without further selection, thereby capturing a broad spectrum of clinical conditions. While the potential for systematic bias remains an inherent limitation in this non-randomized study, we believe our findings reflect real-world clinical practice and offer relevant insights into the safety profile and practical applicability of MRI in aSAH patients under intensive care.

## Conclusion

Compared to existing literature, our study showed a relatively low incidence of systemic, cerebral, and technical complications associated with IHT for brain MRI in patients following aSAH, reflecting a well-established and safe clinical process. Moreover, MRI findings had a notable impact on key therapeutic decisions, supporting the use of MRI-based IHT in selected cases, provided that a thorough risk-benefit assessment is conducted.

## Data Availability

No datasets were generated or analysed during the current study.
